# Mechanism of error-free replication across benzo[*a*]pyrene stereoisomers by Rev1 DNA polymerase

**DOI:** 10.1038/s41467-017-01013-5

**Published:** 2017-10-17

**Authors:** Olga Rechkoblit, Alexander Kolbanovskiy, Hannah Landes, Nicholas E. Geacintov, Aneel K. Aggarwal

**Affiliations:** 1 Department of Pharmacological Sciences, Icahn School of Medicine at Mount Sinai, Box 1677, 1425 Madison Avenue, New York, NY 10029 USA; 2 Department of Chemistry, New York University, 31 Washington Place, New York, NY 10003-5180 USA

## Abstract

Benzo[*a*]pyrene (BP) is a carcinogen in cigarette smoke which, after metabolic activation, can react with the exocyclic *N* ^2^ amino group of guanine to generate four stereoisomeric BP-*N* ^2^-dG adducts. Rev1 is unique among translesion synthesis DNA polymerases in employing a protein-template-directed mechanism of DNA synthesis opposite undamaged and damaged guanine. Here we report high-resolution structures of yeast Rev1 with three BP-*N* ^2^-dG adducts, namely the 10*S* (+)-*trans*-BP-*N* ^2^-dG, 10*R* (+)-*cis*-BP-*N* ^2^-dG, and 10*S* ( − )-*cis*-BP-*N* ^2^-dG. Surprisingly, in all three structures, the bulky and hydrophobic BP pyrenyl residue is entirely solvent-exposed in the major groove of the DNA. This is very different from the adduct alignments hitherto observed in free or protein-bound DNA. All complexes are well poised for dCTP insertion. Our structures provide a view of *cis*-BP-*N* ^2^-dG adducts in a DNA polymerase active site, and offer a basis for understanding error-free replication of the BP-derived stereoisomeric guanine adducts.

## Abstract (web summary)

Benzo[*a*]pyrene (BP) is a carcinogen in cigarette smoke that upon metabolic activation reacts with guanine. Here, the authors present the structures of the translesion DNA synthesis polymerase Rev1 in complex with three of the four possible stereoisomeric BP-*N* ^2^ -dG adducts, which gives insights how Rev1 achieves error-free replication.

## Introduction

Tobacco smoking is directly associated with a majority of lung cancer cases in the United States and is among the few firmly established links between the etiology and the manifestation of this disease^1–4^. Benzo[*a*]pyrene (BP) is one of the most potent and extensively studied carcinogens in cigarette smoke^5^. It is also ubiquitous in the human environment since BP is generated as a result of incomplete combustion of organic matter, such as fossil fuels and wood, and is present in automobile exhaust fumes and charcoal-grilled foods^6–8^. Exposure of animals^9, 10^ and human cells^11^ to BP gives rise to G to T transversion mutations, which are also observed with high frequencies in smoking-related lung cancers^2, 3^ and other malignancies^4^.

**Fig. 1:**
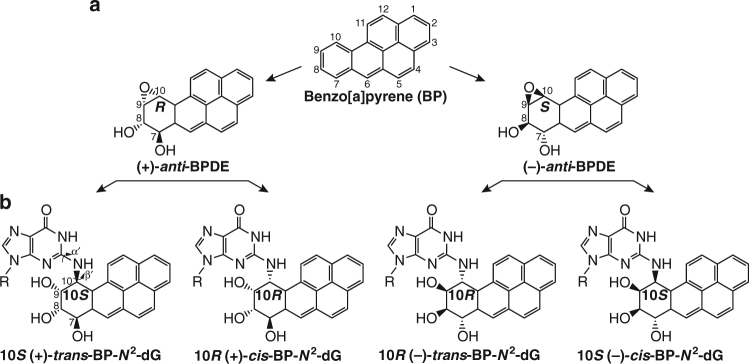
Metabolic activation of benzo[*a*]pyrene (BP) and conformations of stereoisomeric BP-*N* ^2^-dG adducts. **a** Metabolic activation of BP by the cytochrome P450 pathway to (+)-*anti*-BPDE ((+)-7*R*,8*S*-dihydroxy-9*S*,10*R*-epoxy-7,8,9,10-tetrahydrobenzo[*a*]pyrene), and its mirror image enantiomer (−)-*anti*-BPDE ((−)-7*S*,8*R*-dihydroxy-9*R*,10*S*-epoxy-7,8,9,10-tetrahydrobenzo[*a*]pyrene). **b** Stereoisomeric BP-*N* ^2^-dG adducts. These are covalent products derived from the binding of (+)-*anti*-BPDE or (−)-*anti*-BPDE at its C10 position to the *N* ^2^-2′-deoxyguanosine residues in DNA by *trans*- and *cis*- opening of the epoxide ring of BPDE. The (+) and (−) designations denote the mirror image relationships between the absolute configurations of the hydroxyl -OH groups at the C7, C8, and C9 chiral carbon centers. The absolute configuration of the adducts at the C10 carbon atom is 10*S* for the (+)-*trans*-adducts and (−)-*cis*-adducts, and 10*R* for the (−)-*trans*-lesions and (+)-*cis*-lesions. The linkage geometry between the BP and the *N* ^2^ amino group of guanine is defined by the torsion angles α′ and β′ as follows: α′, N1(dG)-C2(dG)-*N* ^2^(dG)-C10(BP), and β′, C2(dG)-*N* ^2^(dG)-C10(BP)-C9(BP). The relative percentage of each adduct formed in human cells is (+)-*trans*, 89%; (−)-*tran*s, 7%; (+)-*cis*, 3%, and (−)-*cis*, 1%^13^

The carcinogenicity of BP derives from its metabolic activation by the cytochrome P450 pathway that generates reactive diol epoxide intermediates that react covalently with DNA^12^. More specifically, the metabolic activation of BP in human cells gives rise to a pair of mirror image BP diol epoxides, the (+)-*anti*-BPDE and (−)-*anti*-BPDE enantiomers (Fig. 1a), each of which reacts predominantly with the exocyclic *N* ^2^ amino group of guanine by *trans*- and *cis*- epoxide ring opening. This leads to four possible stereoisomeric BP-*N* ^2^-dG adducts (Fig. 1b). A minor fraction of adducts are formed by reactions of the diol epoxides with the *N* ^6^-amino groups of adenine^13^. The highly mutagenic and carcinogenic 10*S* (+)-*trans*-BP-*N* ^2^-dG adduct is the predominant steroisomer found in human cells treated with BP (~ 90%)^13^, and is more resistant to removal by the nucleotide excision repair (NER) pathway than the (+)-*cis-*adducts and (−)-*cis-*adducts^14, 15^.

Unrepaired BP-*N* ^2^-dG adducts severely impede high-fidelity DNA polymerases that replicate genomic DNA^16, 17^. Nevertheless, experiments in mammalian cells with site-specifically modified (+)-*trans*-BP-*N* ^2^-dG oligonucleotides embedded in gapped plasmid vectors demonstrate that the adduct is bypassed with ~ 50% efficiency^18, 19^. Such replication obstacles can be handled by specialized, lower fidelity translesion DNA synthesis (TLS) polymerases in the S phase of the cell cycle to ensure continuous progression of the replisome^20^. Alternatively, the damaged DNA site can be skipped to leave a single-stranded DNA gap to be filled in later in the G2 phase of the cell cycle.

Humans possess four Y-family TLS polymerases (Pol η, Pol ι, Pol k, and Rev1) while yeast has two (Pol η and Rev1)^21^. Pol κ and Rev1, can efficiently and preferentially insert the correct cytosine base opposite the BP-*N* ^2^-dG adducts in vitro^22–26^, while Pol η is proficient in incorporating mutation-inducing adenine base^23, 27^, and Pol ι is blocked by the adducts^23^. In addition, the B-family TLS Pol ζ, a “universal extender”, is able to elongate from either the correct or incorrect bases inserted by other polymerases (including Rev1) across the lesion site^28–30^.

In cellular studies, mouse cells deficient in Pol κ have reduced cell survival and accumulate more mutations after exposure to BPDE^31^. In addition, Pol κ was required for recovery from BPDE-induced S-phase checkpoints^32^. Rev1 was found to accumulate in nuclear foci upon exposure to BPDE In human cells^33^, while Pol ζ’s importance was documented in human Nalm-6-MSH + cells^34^. Quantitative assessments of the impact of TLS polymerases on the bypass of ( + )-*trans*-BP-*N* ^2^-dG-containing gapped plasmids were studied by siRNA-induced knockdown of both Pol κ and Pol ζ in human U2OS cells. These experiments revealed that 42% of the TLS bypass events were error-free^29^, suggesting that another TLS polymerase with the ability to insert the correct cytosine base opposite the BP-*N* ^2^-dG adducts was most likely involved.

From in vitro biochemical and structural studies Rev1 is a plausible candidate. The structures of yeast and human Rev1 catalytic cores revealed a novel protein-template-directed mechanism of DNA synthesis^35, 36^, wherein the template dG or an *N* ^2^-dG adduct is evicted from the DNA helix and an arginine makes specific hydrogen bonds with the Watson–Crick (W–C) edge of the incoming dCTP. Rev1 is thus unique among TLS polymerases in its protein-template-directed mechanism of DNA synthesis and its specificity in incorporating C opposite template dG. The Rev1 catalytic activity was shown to be required in vivo for TLS opposite *N* ^2^-dG-derived lesions such as those generated by 4-nitroquinoline-1-oxide (4-NQO)^37^, as well as 1,*N* ^6^-ethenoadenine adducts^38^ and abasic sites^39–41^. In addition, the misincorporation frequencies of Rev1 opposite the 10*S* (+)-*trans*- and 10*R* (*−*)-*trans*-BP-dG adducts are low and similar to those observed with unmodified template dG (~ 10^−2^ to 10^−4^)^24^.

Detailed NMR studies have revealed dramatically different alignments of the covalently attached carcinogen moieties in the different stereoisomeric BP-*N* ^2^-dG adducts in free DNA duplexes in aqueous solution. In case of the *trans*-BP-*N* ^2^-dG adducts, W–C pairing of the modified guanine with the partner cytosine base is maintained^42, 43^, and the BP moiety is situated within the minor groove of the DNA duplex. In the case of the 10*S* (+)-*trans*-adduct^42^, the BP moiety points toward the 5′-end of the modified template strand^42^, while in the case of the 10*R* (−)-*trans*-adduct it points toward the 3′-end^43^. In the case of the 10*R* (+)-*cis-*adducts and 10*S* (−)-*cis-*adducts, the guanine and partner cytosine bases are extruded from the helix, with the hydrophobic BP moiety intercalating within the duplex DNA in place of the displaced G:C base pair^44, 45^. The BP moieties of the two *cis-*adducts are again oriented differently, toward the major and minor grooves of DNA, respectively. Altogether, the stereochemistry of each BPDE adduct determines how it is accommodated in a DNA polymerase active site.

Crystal structures of DNA polymerases with BP-*N* ^2^-dG adducts are limited to the 10*S* (+)-*trans*-stereoisomeric adduct. For example, structures of both A-family Pol I from *Bacillus stearothermophilus* (BF Pol I)^46^ and Y-family human Pol κ^47, 48^ with 10*S* (+)-*trans*-BP-*N* ^2^-dG show that the BP moiety is aligned in the minor groove of the bound DNA, in a manner similar to that anticipated from the NMR structure of the same (+)-*trans*-BP-*N* ^2^-dG in free DNA^42^. By contrast, in structures of Y-family archeal Dpo4 with 10*S* (+)-*trans*-BP-*N* ^2^-dG, the adduct is flipped out the DNA helix, resulting in a non-instructional gap in the template strand^49^.

We present here high-resolution structures of yeast Rev1 with not only the 10*S* (+)-*trans*-BP-*N* ^2^-dG, but also the 10*R* (+)-*cis*-BP-*N* ^2^-dG and 10*S* (−)-*cis*-BP-*N* ^2^-dG adducts as the template bases. Together, these structures provide detailed comparisons of the accommodations of the different BP-*N* ^2^-dG stereoisomeric adducts within the confines of a DNA polymerase active site. Surprisingly, the bulky and hydrophobic BP pyrenyl moiety is entirely solvent-exposed in the major groove of the template–primer DNA helix. This structural alignment is fundamentally different from the ones observed in free DNA as well as previous polymerase-DNA complexes. Furthermore, the structures reveal an opposite orientation of the BP moiety in the 10*S* (+)-*trans*- and 10*R* (+)-*cis*-adducts, imposed by the opposite absolute configurations of the C10 carcinogen-DNA linkage site. We also find that the 10*S* (−)-*cis*- adduct does not mirror the alignment of its 10*R* (+)-*cis*-enantiomer, and thus breaking the “rule” of opposite orientations of the 10*S* and 10*R* stereoisomeric BP-*N* ^2^-dG adducts. Notably, all three complexes are reaction-ready and well poised for dCTP insertion.

## Results

### Preparation of BP-*N*^2^-dG-modified DNA oligonucleotides

Site- and stereo-specifically modified 17-mer DNA oligonucleotides with single (+)-*trans*-BP-*N* ^2^-dG, (−)-*trans*-BP-*N* ^2^-dG, (+)-*cis*-BP-*N* ^2^-dG, or (−)-*cis*-BP-*N* ^2^-dG adducts (Fig. 1b) were generated by the direct synthesis method^50^ using racemic (±)-*anti*-BPDE. Briefly, a 17-mer DNA oligonucleotide with a single G base at the 5th position from the 5′-end (5′-CATCGCTACCACACCCC-3′) was incubated with BPDE. The BP-DNA adducts were separated from the unreacted oligonucleotide and fully hydrolyzed to BP tetraols by HPLC methods (Supplementary Fig. 1a). The mixture of adducted oligonucleotides was than subjected to further separation of components by HPLC methods (Supplementary Fig. 1b and c). To characterize the stereochemistry of the covalently bound BP-*N* ^2^-dG adducts in the different modified oligonucleotides,  ~ 34 μg of each purified sample was subjected to enzymatic hydrolysis to the BP-*N* ^2^-dG nucleoside levels and the circular dichroism (CD) spectra of the hydrolyzed adducts (Supplementary Fig. 1d and e) was compared to the previously described standards^50^.

### Structure determination

We originally sought to co-crystallize the yeast Rev1 polymerase catalytic core with DNA templates containing each of the four stereoisomeric BP-*N* ^2^-dG adducts. We succeeded in co-crystallizing the enzyme with three of the four stereoisomeric adducts, namely the 10*S* (+)-*trans*-BP-*N* ^2^-dG, 10*R* (+)-*cis*-BP-*N* ^2^-dG, and 10*S* (−)-*cis*-BP-*N* ^2^-dG adducts (Fig. 1b **;** Supplementary Fig. 1). The 17-mer templates containing the adducts were paired with a 12-mer primer terminated with 2′,3′-dideoxyguanine (5′-GGGGTGTGGTAG^dd^-3′), and with dCTP as the incoming nucleotide. The complex containing the 10*R* (−)-*trans*-BP-*N* ^2^-dG adduct failed to produce crystals despite the fact that Rev1 has similar dCTP incorporation efficiency opposite both the 10*S* (+)-*trans*-BP-*N* ^2^-dG and 10*R* (−)-*trans*-BP-*N* ^2^-dG adducts^24^. The structure of the 10*R* (+)-*cis*-BP-containing complex was solved by the molecular replacement method using the Rev1 complex with an unmodified DNA and dCTP (PDB ID: 2AQ4)^35^ as a search model and refined at 1.92 Å resolution and R_work/free_ 17.6%/21.4%, respectively. The structure of the 10*S* (+)-*trans*-BP-dG-containing ternary complex was obtained by molecular replacement using the 10*R* (+)-*cis*-BP complex as a search model and refined to 1.85 Å resolution and R_work/free_ 17.0%/20.8%, respectively. The complex containing the 10*S* (−)-*cis*-BP-*N* ^2^-dG was refined to 2.25 Å resolution and R_work/free_ 17.0%/21.6%. The crystal data, together with the data collection and refinement statistics, are summarized in Table 1.

**Table 1:** X-ray data collection and refinement statistics

	10*S* (+)-*trans*-BP-dG	10*R* (+)-*cis*-BP-dG	10*S* (−)-*cis*-BP-dG
*Data collection*			
Space group	P2_1_2_1_2_1_	P2_1_2_1_2	P2_1_2_1_2_1_
Cell dimensions:			
*a*, *b*, *c* (Å)	64.0, 64.9, 130.7	62.5, 180.4, 54.6	63.3, 65.5, 131.7
*α*, *β*, *γ* (°)	90.0, 90.0, 90.0	90.0, 90.0, 90.0	90.0, 90.0, 90.0
Resolution range (Å)^a^	45–1.85 (1.88–1.85)	50–1.92 (1.95–1.92)	45–2.25 (2.29–2.25)
*R*_merge_ (%)	6.6 (45.2)	6.3 (65.0)	10.3 (72.9)
*I*/*σI*	26.3 (2.2)	20.2 (1.4)	18.6 (2.2)
Completeness (%)	99.7 (95.5)	95.3 (92.9)	100 (100)
Redundancy	7.2 (4.2)	5.8 (4.4)	7.4 (6.9)
*Refinement*			
Resolution range (Å)	45–1.85	45–1.92	45–2.25
No. reflections	44,991	43,093	25,424
*R*_work_/*R*_free_	17.0/20.8	17.6/21.4	17.0/21.6
No. atoms			
Protein	3,490	3,481	3,488
DNA	610	615	527
Ligand (dCTP)	28	28	28
Ligand (other)	75	72	59
Ion (Mg^2+^)	4	4	4
Water	340	311	161
B-factors			
Protein	24.0	31.5	37.4
DNA	32.0	38.3	51.6
Ligand (dCTP)	16.8	20.0	28.5
Ligand (other)	39.5	47.9	58.7
Ion (Mg^2+^)	22.8	25.0	31.9
Water	35.4	40.9	41.4
R.m.s. deviations			
Bond length (Å)	0.009	0.010	0.011
Bond angles (°)	1.39	1.39	1.47

^a^Values in parentheses are for highest-resolution shell

### Structure of the 10*S* (+)-*trans*-BP-dG ternary complex

**Fig. 2:**
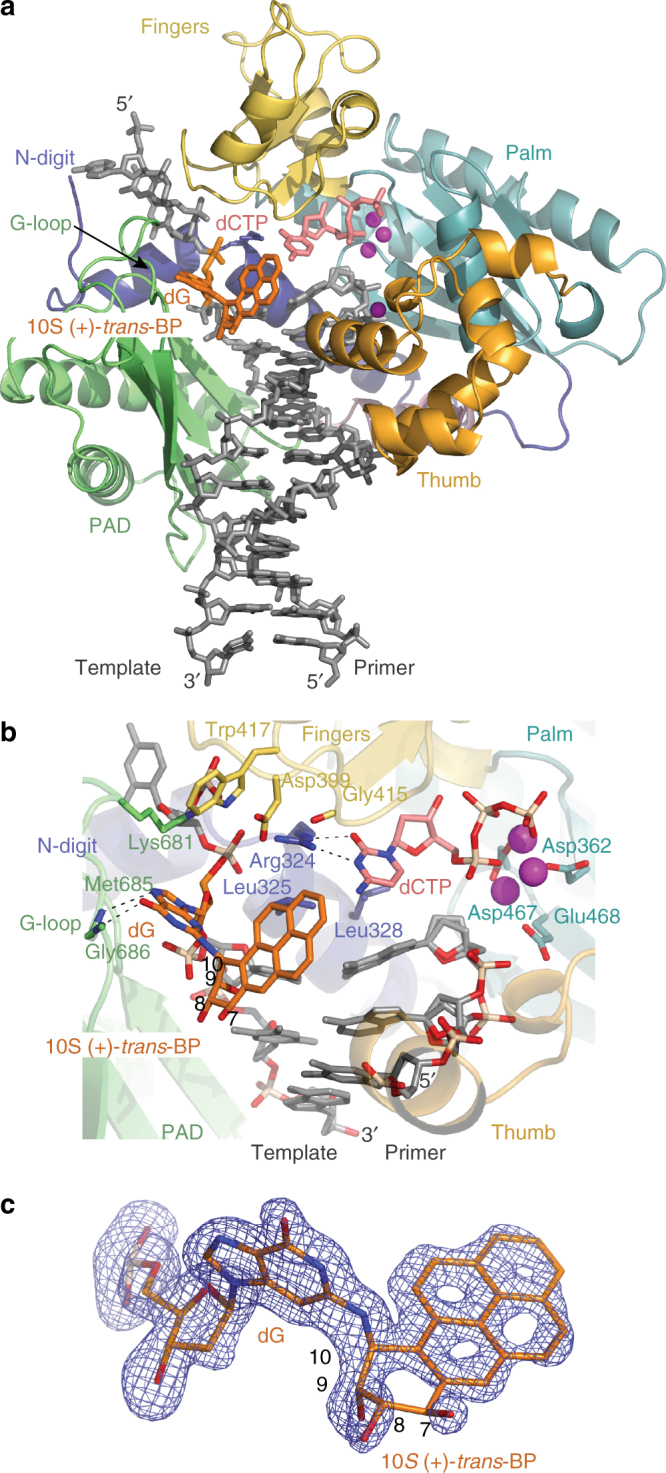
The 10*S* (+)-*trans*-BP-dG-modified Rev1 ternary complex. **a** Overall structure of the complex; the palm, fingers, thumb and PAD domains are shown in cartoon representation in cyan, yellow, light orange, and green, respectively. The linker joining the thumb and the PAD is shown in pink. The α-helical N-digit of Rev1 is shown in dark blue. The DNA template–primer duplex is shown in gray sticks with the template (+)-*trans*-BP-*N* ^2^-dG in orange and incoming dCTP residue in red. The Mg^2+^ ions are represented as magenta spheres. **b** Alignment of the 10*S* (+)-*trans*-BP-*N* ^2^-dG adduct in the Rev1 active site. The 10*S* (+)-*trans*-BP moiety is placed in the solvent-filled space between the PAD and the template–primer DNA helix. Asp362, Asp467, and Glu468 are the catalytic residues. Modified template dG and incoming dCTP partner with segments of Rev1 and not with each other. Arg324 makes hydrogen bonds with the base of dCTP, Leu325 pushes modified template dG out of the DNA helix and Leu328 is partially stacked on top of the 3′-terminal primer base. G-loop residues Met685, Gly686 and Lys681 as well as Trp417, Asp399, and Gly415 interact with the extrahelical template dG. The 3′-terminal and the adjacent residue of the primer strand have double conformations of their phosphate backbone reflecting the mobility of the 3′ terminus in the Rev1 complex. **c** A simulated annealing Fo − Fc omit map (contoured at 3.0σ-level at 1.85 Å resolution and colored in blue) showing the clear electron density for the BP moiety and the modified dG. The dG and the BP C7-OH hydroxyl group are positioned above the BP benzylic ring, while the C9-OH and C8-OH hydroxyl groups are below, thus defining the conformation and the expected stereochemistry of the 10*S* (+)-*trans* BP benzylic ring

Rev1 adopts a very similar conformation to that when it binds unmodified G (Fig. 2a; r.m.s. deviation of 0.76 Å for 433 Cαs). That is, the enzyme embraces the template–primer DNA with its palm (residues 356–365, 438–536), fingers (residues 366–437), and thumb (537–603) domains as well as the PAD (polymerase associated domain; residues 621–738), which is unique to Y-family polymerases (Fig. 2a)^35^. An α-helical substructure at the N terminus, an N-digit (residues 305–355) augments this embrace and makes critical interactions with the incoming dCTP and the templating base. The active site residues (Asp362, Asp467, and Glu468) are located on the palm domain to catalyze the nucleotidyl transfer reaction (Fig. 2b). The fingers domain interacts with the incoming dCTP and the 5′-template overhang, while an extra long loop of the PAD, a G loop, interacts with the base of the modified guanine. The thumb and the PAD are positioned on opposite sides of the DNA duplex and are connected by a long helical linker (Fig. 2a). The incoming dCTP does not pair with templating 10*S* (+)-*trans*-BP-dG; instead, Arg324 (from the N-digit) acts as a “surrogate” residue and makes a set of complementary hydrogen bonds with the cytosine base of the incoming dCTP (Fig. 2b) as observed previously in the unmodified structure^35^.

**Fig. 3:**
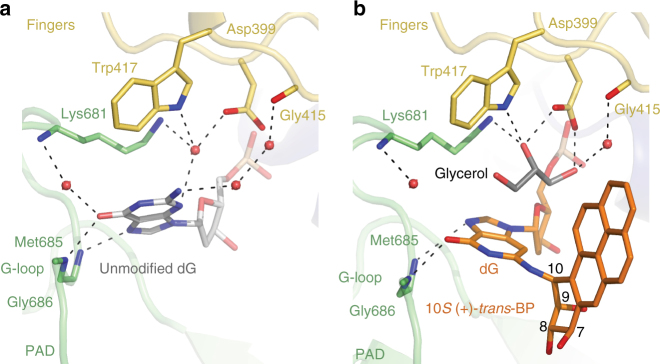
A close up view of the unmodified dG and 10*S* (+)-*trans*-BP-*N* ^2^-dG adduct contacts with Rev1. **a** The template dG in the unmodified complex (PDB ID: 2AQ4)^35^. The backbone amines of Met685 and Gly686 form direct hydrogen bonds with the base of the dG. Side chains of residues Asp399, Trp417, Lys681 and Gly415 and the backbone amine of Lys681 form water-mediated contacts with the base. **b** The 10*S* (+)-*trans*-BP-*N* ^2^-dG contacts with Rev1. The modified dG maintains hydrogen bonds between the N7 and *O* ^6^ atoms at its “Hoogsteen edge” and the main-chain amides of Met685 and Gly686 of the G loop. However, water-mediated contacts with Asp399, Trp417, Lys681, and Gly415 are lost. A molecule of glycerol (shown in gray) appears to take the place of some of the water molecules observed in the unmodified structure (**a**)

The modified dG is evicted from the DNA helix by Leu325 from the N-digit (Fig. 2b). The base rotates  ~ 90° away from the minor groove so that the *N* ^2^ amino group now faces the major groove of the DNA duplex (instead of its usual placement in the minor groove). Consequently, the BP moiety is located in the major groove of the DNA. The stereochemistry of the BP moiety is well defined in the 1.85 Å electron density map and reveals an orientation that is almost perpendicular to the plane of the guanine base (torsion angle β′ C2(dG)-*N* ^2^(dG)-C10(BP)-C9(BP) is −75.9°) (Fig. 2c and Supplementary Fig. 2a). The low average B-factors for atoms of the BP and the modified dG moieties (32.6 and 20.8 Å^2^, respectively) are close in value to the B-factors of the atoms of protein-bound portion of the DNA duplex (24.5 Å^2^) and indicate the well-ordered alignment of the adduct. Interestingly, the BP pyrenyl ring system points towards the 5′-direction of the modified strand (Fig. 2b and Supplementary Fig. 2a). This is made possible by the modified dG base tilting by ~ 44° in the 3′-direction to create room for the BP moiety to extend in the opposite direction, as shown in Fig. 3a, b. Furthermore, the BP moiety is rotated away from the W–C edge of dG, wherein the benzylic ring torsion angle α′ (N1(dG)-C2(dG)-*N* ^2^(dG)-C10(BP) is 163.8° (Figs. 2b, c). The hydroxyl groups at the C9 (C9-OH) and C8 (C8-OH) of the BP face away from the W–C edge of dG. The overall conformation of the BP-base linkage is in the most energetically favorable region (α′ = 180 ± 40° and β′ = -90 ± 40°) as calculated for an isolated (+)-*trans*-BP-dG nucleoside^51^.

Despite the tilt in the dG base to make room for the BP moiety, it maintains (as in the unmodified Rev1 complex structure) hydrogen bonds between the N7 and *O* ^6^ atoms at its “Hoogsteen edge” and the main-chain amides of Met685 and Gly686 of the G loop (Fig. 3a, b). However, a number of water-mediated contacts are lost, including a water-mediated interaction between the N3 atom and the side chains of Asp399, Trp417, and Lys681, a water bridge between the *O* ^6^ atom and the backbone NH of the Lys681, as well as a two-water molecule bridge between the *N* ^2^ group and the backbone carbonyl of Gly415. Interestingly, a molecule of glycerol (shown in gray on Fig. 3b) appears to take place of some of the water molecule observed in the unmodified structure (Fig. 3a).

### Structure of the 10*R* (+)-*cis*-BP-dG ternary complex

**Fig. 4:**
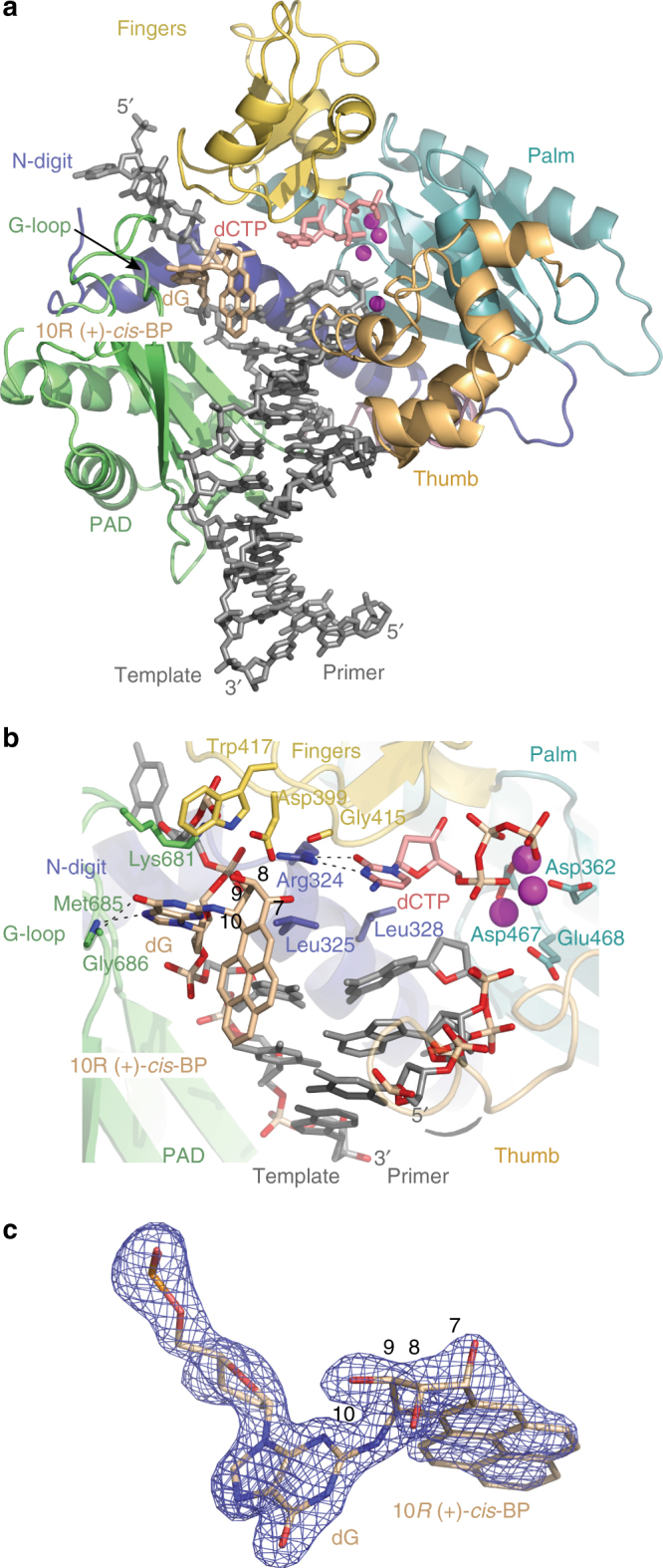
The 10*R* (+)-*cis*-BP-*N* ^2^-dG–modified Rev1 ternary complex. **a** Overall structure of the complex. The coloring scheme and the details shown are as in Fig. 2. The template 10*R* (+)-*cis*-BP-*N* ^2^-dG is in beige. **b** Alignment of the 10*R* (+)-*cis*-BP-*N* ^2^-dG adduct in the Rev1 active site. The 10*S* (+)-*trans*-BP moiety is positioned in the solvent-filled space between the PAD and the template–primer DNA helix. **c** A simulated annealing Fo−Fc omit map (contoured at 3.5σ at 1.92 Å resolution and colored in blue) showing clear density for the BP moiety and the modified dG. The dG as well as the C9-OH and C8-OH hydroxyl groups are oriented below the BP benzylic ring, while the C7-OH is above the ring. This confirms the expected stereochemistry of the 10*R* (+)-*cis* BP benzylic ring

**Fig. 5:**
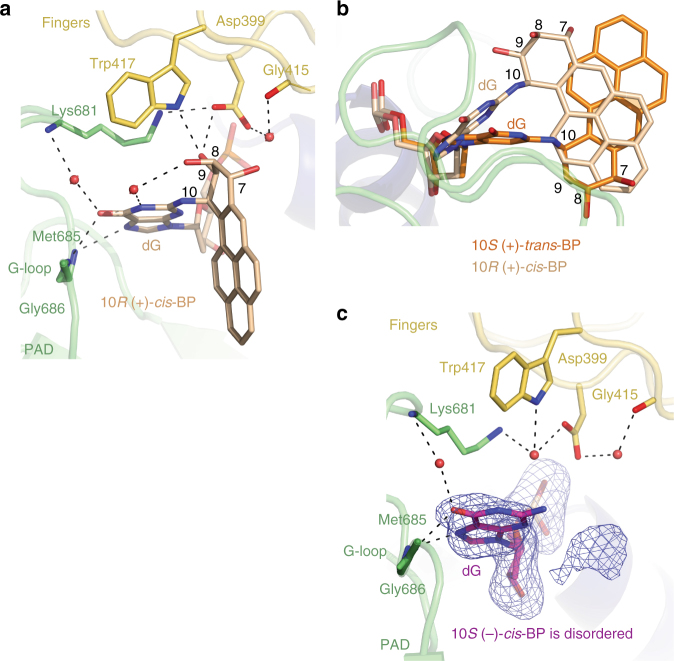
A close up view of the 10*R* (+)- and 10*S* (−)-*cis*-BP-*N* ^2^-dG adducts within Rev1 and comparisons with the 10*S* (+)-*trans-*BP structure. **a** Contacts of the 10*S* (−)-*cis*-BP-*N* ^2^-dG adduct with the Rev1 protein. The BP-modified dG forms hydrogen bonds between the N7 and *O* ^6^ atoms at its Hoogsteen edge and the main-chain amides of Met685 and Gly686 of Rev1. There is also a water-mediated bond between the *O* ^6^ of the base and the peptide amide of Lys681. The C9-OH of the BP moiety forms hydrogen bonds with the side chains of Trp417 and Asp399 and the C8-OH group of the BP is bridged to the N1 of the dG base through a water molecule. **b** Comparison of the 10*R* (+)-*cis*-BP-*N* ^2^-dG and 10*S* (+)-*trans*-BP adduct alignments. The 10*R* (+)-*cis*-BP-dG (shown in beige colored sticks) in the complex with Rev1 is superpositioned on the 10*S* (+)-*trans*-BP adduct structure (shown in orange sticks); the structures are aligned by Rev1 protein chains. The 10*S* ( + )-*trans*-BP and 10*R* (+)-*cis*-BP- moieties are oriented in the opposite directions. However, they occupy approximately the same physical space in the Rev1 active site. **c** A simulated annealing Fo−Fc omit map (contoured at 3.0σ-level at 2.24 Å resolution and colored in blue) showing clear electron density of the (−)-*cis*-BP-modified dG base (magenta sticks). Slight change in the position of the modified dG base (as compared to the unmodified dG as shown in Fig. 3a) places the N3 atom 3.6 Å away from the water molecule that is firmly coordinated via the side chains of Asp399, Trp417, and Lys681 -too far for a hydrogen bond formation. Despite the reasonably high 2.24 Å resolution, the electron density map for the BP moiety is not well defined and the conformation of the adduct cannot be established unambiguously. However, there is a residual electron density of the BP moiety below the dG base

In contrast to the 10*S* (+)-*trans*-BP-dG ternary structure, the BP moiety is directed towards the 3′-end of the template strand (Figs. 4a, b). This likely reflects the fact that the absolute configuration of the C10 DNA linkage of the 10*R* (+)-*cis*-BP-dG adduct is a mirror of the 10*S* (+)-*trans*-BG-dG adduct (Fig. 1b). The conformations of the 10*R* (+)-*cis*-BP and modified dG moieties are well ordered as indicated by the 1.92 Å electron density map (Fig. 4c and Supplementary Fig. 2b) and low B-factors (39.9 and 24.9 Å^2^, respectively) that are close in value observed for the Rev1-bound portion of the DNA (33.0 Å^2^). The torsion angle β′ (C2(dG)-*N* ^2^(dG)-C10(BP)-C9(BP)) is 111.8° (Fig. 4c), as compared to −75.9° in the 10*S* (+)-*trans*-BP-dG ternary structure (Fig. 2c). Consequently, the benzylic ring of the 10*R* (+)-*cis*-BP residue is now above the guanine base in close proximity to the fingers domain of Rev1 (Fig. 4b). Moreover, the base of the modified dG remains untilted, and many of the direct and water-mediated hydrogen bonds observed in the structure with unmodified dG are present (Figs. 3a and 5a). This includes hydrogen bonds between the N7 and *O* ^6^ atoms at the Hoogsteen edge of the base and the main-chain amides of Met685 and Gly686 (Fig. 5a), and water-mediated bonds between the *O* ^6^ of the base and Lys681. Notably, the C9-OH displaces the highly coordinated water molecule and forms direct hydrogen bonds with the Rev1 side chains of Asp399 and Trp417 (Fig. 5a). There is also a water-mediated intermolecular bridge between the N1 of dG and the C8-OH hydroxyl of the BP moiety.

Although, the pyrenyl ring system points in opposite directions in the 10*S* (+)-*trans*- and 10*R* (+)-*cis*-BP-dG adducts, they occupy relatively the same physical space in the Rev1 active site, between the PAD and the fingers domain (Fig. 5b). Also, in both cases the pyrenyl ring system is directed away from the W–C edge of the dG (torsion angle α′ = 175.3°). However, the C9-OH and C8-OH hydroxyl groups are oriented toward the W–C edge of the dG residue due to the intrinsic stereochemistry of the *cis*-adducts that places the C9 and C8 hydroxyls on the same side of the benzylic ring as the guanine base (Fig. 4c). This conformation of the 10*R* (+)-*cis*adduct is within the most energetically favorable region calculated for this nucleoside (α′ = 185 ± 35° and β′ = 100 ± 30°)^51^. Otherwise, the overall structures of the 10*R* (+)-*cis*-BP-dG-modified and 10*S* (+)-*trans*-BP-dG-modified complexes are similar (r.m.s. deviation = 0.97 Å for 433Cαs) (Figs. 2a and 4a).

### Structure of the 10*S* (−)-cis-BP-dG ternary complex

In contrast to the 10*S* (+)-*trans*-BP-dG and 10*R* (+)-*cis*-BP-dG structures described above, the electron density for the BP moiety is not well defined in the 10*S* (−)-*cis*-BP-dG structure (Fig. 5c). However, the electron density for the dG base is well defined and shows it in an untilted orientation, similar to the one observed in the 10*R* (+)-*cis*-BP complex. Consequently, there is no room for the bulky BP pyrenyl ring system to reside above the guanine base to mirror the 10*R* (+)-*cis*-BP adduct alignment. Thus, the 10*S* (−)-*cis*-BP pyrenyl ring system most likely points toward the 3′-direction of the modified strand, where there is enough space for an alignment similar to the one acquired by the 10*R* (+)-*cis*-BP moiety (Fig. 5a). This assumption is consistent with the residual electron density observed below the guanine base. Such an orientation would place the 10*S* (−)-*cis*-BP-dG into a less energetically favorable conformational region (α′ = −10 ± 40° and β′ = −100 ± 30°)^51^ with the dG N1 edge and benzylic BP edge closer to each other. The higher energetic cost of such orientation stems from the stereo crowding between the N1 edge of guanine and the bay region of the aromatic BP pyrenyl moiety. While computationally feasible, BP-dG conformations in this region of the potential energy surface have hitherto not been detected experimentally. Thus, this structure provides experimental evidence that the BP moieties of the 10*R* (+)-*cis-*BP-dG and 10*S* (−)-*cis*-BP-dG adduct pair can be oriented the same way and not in an approximate mirror arrangement. The observed disorder in the electron density map in the case of the 10*S* (−)-*cis*-BP moiety is, probably, due to the BP residue sampling the large number of possible conformations in a search for a more favorable alignment within the Rev1 complex crystal.

### Metal ions in the active site

**Fig. 6:**
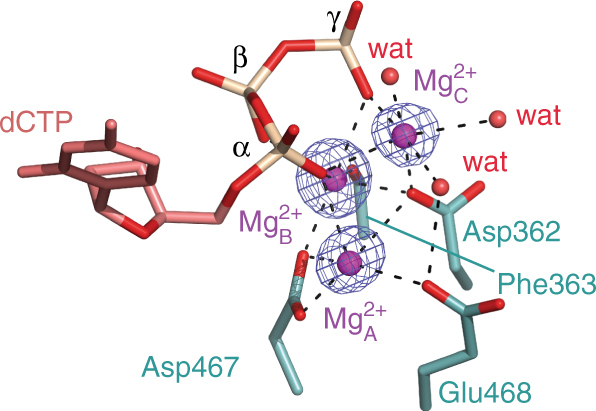
The third Mg^2+^ ion (Mg^2+^_C_) at the Rev1 active site. The first Mg^2+^, cation A, is coordinated by the invariant Asp362, Asp467, and Glu468 residues. The second Mg^2+^_B_, is chelated by the phosphate groups of the incoming dCTP and the main-chain carbonyl of Phe363. The third, Mg^2+^_C_, has six ligands and is coordinated by the two non-bridging oxygen atoms of the α- and γ-phosphates of dCTP, a carboxylate oxygen of Asp362 and three waters. The simulated annealing Fo − Fc map with Mg^2+^_C_ omitted contoured at 7.0σ-level at 1.85 Å resolution is shown in blue. Hydrogen bonds and metal-ion coordination are shown as dashed lines

In all three structures, there are three Mg^2+^ ions (Mg^2+^_A_, Mg^2+^_B_, and Mg^2+^_C_) in the active site (Fig. 6). Mg^2+^_A_, Mg^2+^_B_ are analogous to the two metal ions present in high fidelity and TLS polymerases^52^. Mg^2+^_C_ has an octahedral geometry with short ligation distances and is coordinated by the two non-bridging oxygen atoms of the α- and γ-phosphates of dCTP, a carboxylate oxygen of Asp362 and three waters. A similar Mg^2+^_C_ ion has been observed in the active site of high-fidelity Pol δ^53^, and may facilitate dCTP binding to the active site and assist in the leaving of the pyrophosphate. This Mg^2+^_C_ ion differs from the transient product-associated ion that binds between the α- and β-phosphates of dNTP during phosphodiester bond formation in Pol η^54^ and Pol β^55^ complexes. A fourth Mg^2+^ ion (Fig. 2a) is observed bridging the phosphate group of the primer base next to the primer terminus and the loop of the thumb domain. An analogous entity has been previously assigned as a water molecule in the yeast Rev1 structures^35, 56^ and as a Mg^2+^ ion in the human Rev1 complex^36^.

## Discussion

We show here that Rev1 is remarkably well-adapted for the error-free bypass of 10*S* (+)-*trans*-BP-*N* ^2^-dG, 10*R* (+)-*cis*-BP-*N* ^2^-dG, and 10*S* (−)-*cis*-BP-*N* ^2^-dG stereoisomeric adducts. The ability of Rev1 to evict these bulky adducts from the DNA helix and to position Arg324 as the surrogate protein template residue allows for the incorporation of a correct C opposite these stereoisomers, consistent with the previous in vitro studies^24^. Strikingly, the BP moiety in each case is pushed into the more capacious major groove side of the DNA, where it is solvent-exposed. This positioning of the BP moiety in the DNA major groove differs fundamentally from the configuration in free DNA. From solution NMR studies of BP lesions within B-DNA^57^, the 10*S* (+)-*trans*-adducts and 10*R* (−)-*trans*-adducts reside in the minor groove, while the 10*R* (+)-*cis*-adducts and 10*S* (−)-*cis*-adducts adopt an intercalative conformation where the pyrenyl ring system displaces the dG:dC base pair and assumes an intercalated conformation^44, 45^. Moreover, in free DNA, one face of the hydrophobic pyrenyl ring system is shielded from solvent by van der Waals interactions with the DNA backbone (10*S* (+)-*trans*-adducts and 10*R* (−)-*trans*-adducts), while both faces are shielded by stacking between adjacent bases within the DNA helix ((+)-*cis*-adducts and 10*S* (−)-*cis*-adducts). By contrast, in the Rev1 active site, both faces of the hydrophobic pyrenyl ring system are solvent-exposed (Fig. 2b). The Rev1 PAD and fingers domain, as well the DNA sugar-phosphate backbone, are too far away to interact directly with the pyrenyl ring system in all three structures.

Although the 10*S* (+)-*trans*-BP-*N* ^2^-dG adduct differs in how it is accommodated in the Rev1 active site versus free DNA, the torsion angles α′ and β′ defining the local conformation of the carcinogen-DNA linkage site (Fig. 1b) are similar in both cases and are in the most favorable range for a 10*S* (+)-*trans*-BP-dG-modified nucleoside, namely α′ = 180 ± 40°, β′ = -90 ± 40°^51^. This similarity in α′ and β′ torsion angles partly underlies the fact that the BP pyrenyl ring system points towards the 5′ end of the modified strand in both the Rev1 active site and in free DNA (Fig. 2b). The 10*S* ( + )-*trans*-BP-*N* ^2^-dG adduct in this favorable energy region has also been captured in the active sites of several other DNA polymerases, including BF Pol I^46^, archeal Dpo4^49^, and human Pol κ^47, 48^. However, the overall adduct alignments in these complexes are strikingly distinct from the one in the Rev1 complex. In BF Pol I and Pol κ, the BP moiety lies in the minor groove and is directed toward the 5′ of the template strand, adopting the conformations similar to the ones observed in the free DNA in solution^44, 57^. In these structures, the BP moiety is protected from the solvent with one face of the pyrenyl ring system packed against the protein and the other against the DNA backbone. Whereas, in Dpo4, the BP-dG adduct is flipped out the DNA helix toward the minor groove and inserted into a solvent-protecting cleft between the fingers and the PAD domains, resulting in a non-instructional gap in the template strand. Interestingly, the structure of a base excision repair polymerase β in complex with the *N* ^2^-dG adduct of the benzo[*c*]phenanthrene (BPh) diol epoxide also shows the adduct in a similar conformation as in free DNA^58^.

The 10*R* (+)-*cis*-BP-dG and 10*S* (−)-*cis*-BP-dG ternary complex structures presented here show the *cis*-adducts in the confines of a DNA polymerase active site. The intercalative conformation of these adducts in free DNA has been ascribed to the C9-OH and C8-OH hydroxyl groups pointing inward toward the DNA backbone, which lends to an already crowded situation in the minor groove of DNA^51^. We show here that the *cis*-adducts can occupy the major groove when bound to Rev1 (Fig. 5a, c) so that the C9-OH and C8-OH hydroxyl groups are far-removed from the neighboring bases. The torsion angles α′ and β′ for the 10*R* (+)-*cis*-BP-dG carcinogen-base linkage in the Rev1 active site are 175° and 112°, respectively, as compared to 160° and 136° in the free DNA^44^. Thus, despite very different conformations, the torsion angles are in a similar range, as well as within the most stable potential energy surfaces of the 10*R* (+)-*cis*-BP-modified nucleoside^51^. This supports the idea that the intercalative conformation in free DNA is dictated less by the BP-dG linkage site α′ and β′ torsion angles and more by changes in DNA backbone torsion angles in the nucleotides adjacent to the adduct residues. Curiously, the BP moiety appears to adopt multiple conformations in our 10*S* (−)-*cis*-BP-dG ternary complex structure. These multiple conformations may stem from the fact that they appear to be centered on less favorable portion of the potential energy surface, namely α′ = −10 ± 40°^51^. Indeed, this allows for the BP pyrenyl aromatic ring system of the 10*S* (−)-*cis* adduct to point away from the finger domain of Rev1 and toward the 3′-end of the template as observed in the case of the 10*R* (+)-*cis*-BP stereoisomeric adduct. By contrast, when the 10*S* (−)-*cis*-BP-dG adduct structure is modeled with the more favorable torsion angles of 185 ± 35° and −100 ± 30°, the pyrenyl rings point into opposite directions resulting in severe steric clashes with the protein backbone of Ser398 and Asp399 and side chains of Asp399 and Trp417.

Despite strong biochemical and structural data demonstrating the ability of Rev1 to accommodate and bypass BP-dG adducts in vitro, the role of Rev1 in BP-dG bypass in vivo remains to be fully realized. In addition to its catalytic function, Rev1 also has a scaffolding function whereby its C-terminus can coordinate TLS via interactions with other TLS polymerases^59–62^. In budding yeast, the Rev1 catalytic activity has been shown to be required in vivo for TLS across *N* ^2^-dG-derived lesions such as 4-nitroquinoline-1-oxide (4-NQO)^37^, as well as 1,N^6^-ethenoadenine adducts^38^, but in human cells the scaffolding function of Rev1 appears to play an important role^63, 64^. The structures of the catalytic cores of human and yeast Rev1 are very similar^36^. The main difference is the presence of a hydrophobic “flap” on the pocket that holds the template G base, which if anything, appears to facilitate BP-*N* ^2^-dG lesion bypass in vitro^24^ (Supplementary Fig. 3). Taken together, the structures we present here provide a basis for further studies to dissect the relative contributions of the Rev1 catalytic and scaffolding functions in BP-*N* ^2^-dG lesion bypass.

## Methods

### Preparation of the BP-*N*^2^-dG-modified 17-mer DNA templates

Site- and stereo-specifically modified 17-mer DNA oligonucleotides with single (+)-*trans*-anti-BP-*N* ^2^-dG, (−)-*trans*-anti-BP-*N* ^2^-dG, (+)-*cis*-anti-BP-*N* ^2^-dG, or (−)-*cis-*anti-BP-*N* ^2^-dG lesions were generated by a direct synthesis method^65^ using racemic ( ± )-*anti*-BPDE obtained from the National Cancer Institute Carcinogen Reference Standard Repository (currently available from MRIGlobal Chemical Carcinogen Repository). The procedures used for the synthesis and adduct purification and the methods of verification of adduct stereochemistry were similar to those described previously for an 11-mer sequence^50^. Briefly, ~ 83.8 mg (~ 2500 OD_260_ units) of the 17-mer oligonucleotide 5′-CATCGCTACCACACCCC-3′ (Integrated DNA Technologies) were dissolved in 10 ml of triethylamine acetate (TEAA)-sodium acetate pH 11 buffer. Racemic BPDE dissolved in 2 ml of tetrahydrofuran was added to a DNA solution in the molar ratio of BPDE to DNA 2:1. The mixture was allowed to react in darkness at + 4 °C temperature with gentle mixing for one week to ensure complete reaction. The products in the reaction mixture were separated by HPLC using a PRP-1 polymeric HPLC preparative column (Hamilton). The HPLC conditions employed were a linear 10–30% acetonitrile/50 mM TEAA pH 7.0 buffer gradient in 60 min. A UV detector (wavelength set at 254 nm) and a fluorescence detector (emission wavelength = 400 nm, excitation wavelength = 343 nm) were used to monitor the elution profiles. A typical elution profile is shown in Supplementary Fig. 1a. The unmodified oligonucleotide elutes first (at ~ 16–24 min), followed by the crude mixture of BP-DNA adducts dominated by BP-*N* ^2^-dG adducts (collected at ~ 26–35 min) and by a mixture of minor BP-*N* ^6^-dA adducts (at ~ 35–45 min), which are characterized by higher fluorescence intensities than the BP-*N* ^2^-dG adducts. Fully hydrolyzed BP tetrol (BPT) was washed from the column with 80% acetonitrile (at ~ 50 min). After collection, the BP-*N* ^2^-dG adduct mixture was vacuum dried, re-dissolved in water and further purified by HPLC in several injection steps with 10–20% acetonitrile/50 mM TEAA pH 7.0 buffer gradient in 60 min on C18 (ACE) column Supplementary Fig. 1b. Each elution peak was collected separately, combined with the corresponding peak from the other injection steps and vacuum dried. The purity check of Peak1 is shown in Supplementary Fig. 1c. Furthermore, the BP-*N* ^2^-dG adducts were desalted with SlideSlide-A-Lyzer Dialysis Cassettes (ThermoFisher). To characterize the stereochemistry of the BPDE linkage, ~ 34 μg of each purified adducted 17-mer was subjected to enzymatic hydrolysis to the BP*-* *N* ^2^-dG-nucleoside levels. The stereochemistry was assigned based on the circular dichroism (CD) spectra of the hydrolyzed adducts (Supplementary Fig. 1d, e) and the previously described standards^50^. The BP-*N* ^2^-dG 17-mer yields were ~ 1.0 mg in the case of the (+)-*trans*-BP-modified, ~ 0.23 mg of the (−)-*trans*-BP-modified, ~ 0.84 mg of the (+)-*cis*-BP-modified, and ~ 0.77 mg of the (−)-*cis-*BP-modified oligonucleotides.

### Expression and purification of yeast Rev1 protein

The *Saccharomyces cerevisia*e catalytic core Rev1 protein (residues 297–746) was expressed in Escherichia coli BL21 (DE3) codon Plus RIL (Stratagene) cells as an N-terminally tagged glutathione S-transferase (GST) fusion protein. The cells were grown in Luria-Bertani (LB) medium at 37 °C and expression of the fusion protein induced by the addition of 0.1 mM isopropyl-β-D-1-thiogalactopyranoside (IPTG) followed by overnight incubation at 18 °C. The GST-Rev1 fusion protein was purified from bacterial lysate by affinity chromatography using a glutathione-Sepharose column. The GST tag was next cleaved on the resin with PreScission protease and Rev1 (297–746) was eluted from the column as described previously^35^. The protein was further purified by chromatography on HiTrap Heparin column, following by passage through a Superdex 75 column (GE Healthcare). The protein was concentrated to  ~ 12 mg ml^−1^ in 25 mM tris (pH 8.0), 250 mM NaCl, and 2 mM tris(2-carboxyethyl) phosphate (TCEP) and stored in aliquots at −80 °C.

### Crystallization

The crystals of the yeast Rev1 ternary complexes containing 10*S* (+)-*trans*-BP-*N* ^2^-dG, 10*R* (+)-*cis*-BP-*N* ^2^-dG, or 10*R* (−)-*trans*-BP-*N* ^2^-dG 17-mer templates and 12-mer complementary primer terminated with 2′,3′-dideoxyguanine (5′-GGGGTGTGGTAG-3′) in the presence of dCTP were obtained by a hanging drop method against a reservoir solution containing 0.25 M sodium citrate pH 6.0 buffer and 15–20% PEG3350. Briefly, the template–primer DNAs were annealed and mixed with Rev1 protein in a 1.2:1 molar ratio to a final complex concentration of 0.11 mM in 22 mM tris (pH 8.0), 160 mM NaCl, 1.5 mM TCEP, 10 mM MgCl_2_, and 10 mM dCTP. The complexes were incubated at room temperature for 10 min and then centrifuged at 10,000 r.p.m. for 7 min at 4 °C. Crystallization drop was formed by mixing 1 μL of the complex with 1 μL of the reservoir solution and the crystals were grown at 20 °C. The complex containing 10*R* (−)-*trans*-BP-*N* ^2^-dG adducted oligonucleotide failed to produce crystals. The crystals were cryoprotected in the reservoir solution supplemented with 24% PEG3350 and 20% glycerol and flash frozen in liquid nitrogen for X-ray data collection. Several rounds of microseeding were necessary to produce the large diffraction-quality crystals.

### Structure determination and refinement

The X-ray diffraction data were collected at the NSLSX25 beam line at the Brookhaven National Laboratory. The data were processed and scaled using the HKL2000 suite^66^. The structure of the 10*R* ( + )-*cis*-BP-dG complex was solved by the molecular replacement method (Phaser)^67^ in the CCP4 program package^68^, using a previously published structure with an unmodified dG and incoming dCTP, PDB ID 2AQ4^35^ (with a different DNA sequence) as search model. The model building, including substitution of the DNA sequence, was finished manually in Coot^69^ based on the electron density maps calculated in REFMAC^70^ in the CCP4 suite. The resulting model was refined in REFMAC in space group P 2_1_2_1_2 with *a* = 63 Å, *b* = 180 Å, *c* = 55 Å unit cell to 1.92 Å and R_work/free_ 18.3%/21.8%, correspondently. The placement of the BP and modified dG moieties was verified using the simulated annealing omit maps calculated in Phenix^71^ with the (+)-*cis*-BP-*N* ^2^-dG omitted from the models before heating them to 2000 K and then slowly cooling them. The refined 10*R* (+)-*cis*-BP-dG structure includes Rev1 residues 307–738, nucleotides 2–17 for the template strand, nucleotides 1–12 for the primer strand, incoming dCTP, 4 Mg^2+^ ions, and 311 water molecules. The 3′-terminal and the adjacent residue of the primer strand have double conformations of their phosphate backbone reflecting the mobility of the 3′ terminus in Rev1 complex. The structure of 10*S* (+)-*trans*-BP-dG ternary complex was obtained by molecular replacement using the 10*R* (+)-*cis*-BP complex as a search model. The placement of the 10*S* (+)-*trans*-BP*-* *N* ^2^-dG adduct was verified using the simulated annealing omit maps as described above. The model was refined to 1.85 Å in space group P 2_1_2_1_2_1_; *a* = 64 Å, *b* = 65 Å, *c* = 131 Å and R_work_/_free_ 17.7%/21.7%, respectively.

### Data availability

Atomic coordinates and structure factors have been deposited in the Protein Data Bank under accession codes 5WM1, 5WM8 and 5WMB for the 10*S* (+)-*trans*-, 10*R* (+)-*cis*- and 10*S* (−)-*cis*-BP-dG-containing Rev1 ternary complexes, respectively. Other data are available from the corresponding author upon reasonable request.

## Electronic supplementary material

Supplementary Information
