# Supplementary material for: Mechanism of error-free replication across benzo[a]pyrene stereoisomers by Rev1 DNA polymerase
Source: Nat Commun. 2017 Oct 17;8:965. doi: 10.1038/s41467-017-01013-5 (PMC5645340; doi:10.1038/s41467-017-01013-5)
Supplement: Supplementary file 1 — Supplementary Information [file 41467_2017_1013_MOESM1_ESM.pdf]

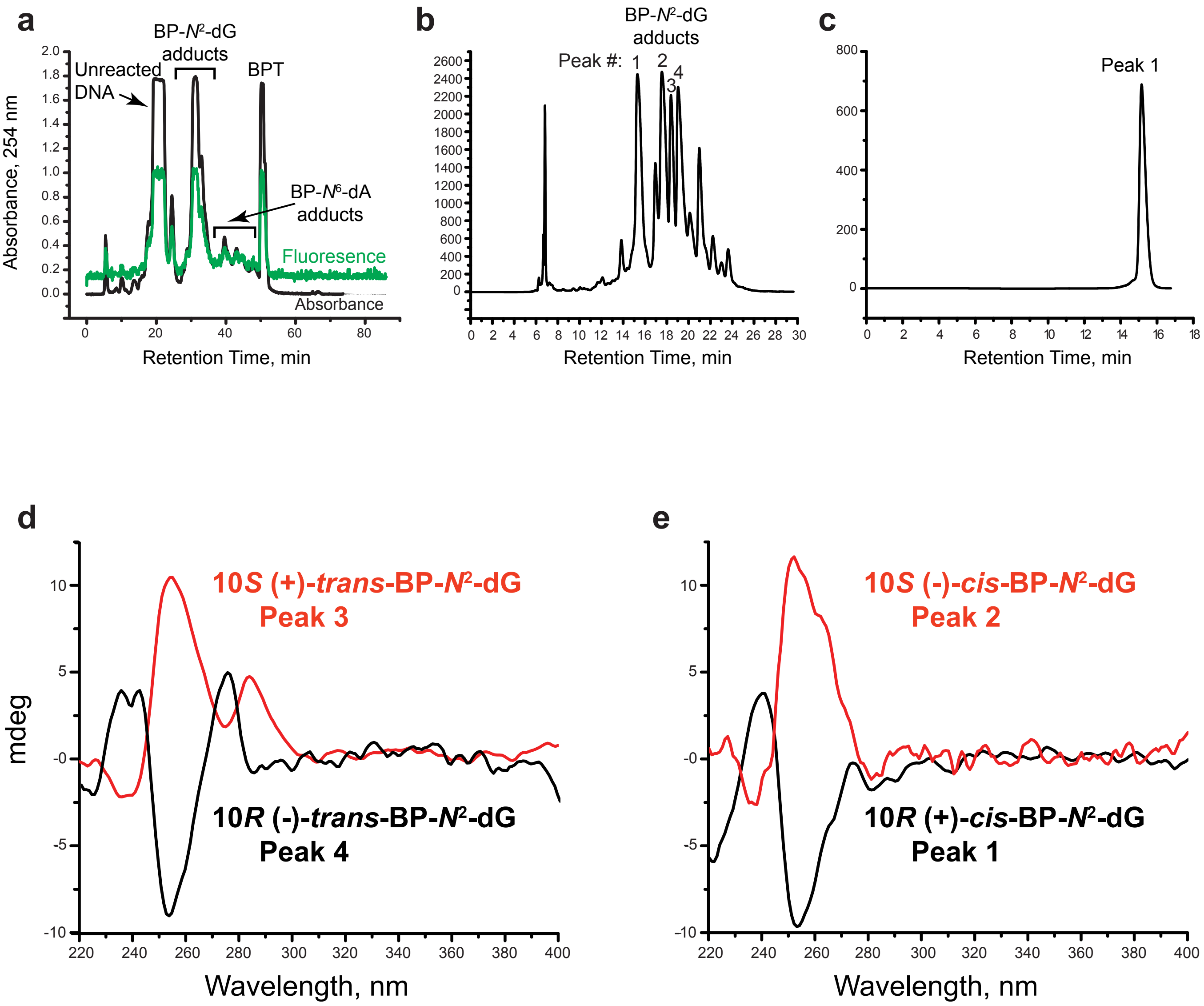


Supplementary Figure 1

**Supplementary Figure 1. Purification and assignments of the stereochemistry of the BP-*N*²-dG-modified 2'-deoxyribooligonucleotides.** (**a**) Crude separation of BP-DNA adducts by HPLC after incubation of the 17-mer DNA oligonucleotide 5′-CATCGCTACCACACCCC with racemic (±)-BPDE. The unmodified oligomer elutes first (at ~16-24 min), followed by a crude mixture of BP-DNA adducts dominated by BP-$N^2$-dG adducts (collected at ~26-35 min), and by a mixture of minor BP-$N^6$-dA adducts (at ~35-45 min), which have higher fluorescence intensity than BP-$N^2$-dG adducts. Fully hydrolyzed benzo[*a*]pyrene tetraol (BPT) was washed from the column with 80% acetonitrile (at ~50 min). (**b**) 2$^{nd}$ HPLC purification step to separate BP-$N^2$-dG adducts Peaks # 1-4. (**c**) Purity check of Peak 1. (**d**) and (**e**) Circular dichroism (CD) spectra of enzymatically digested to BP-$N^2$-dG -nucleobase level *trans*- and *cis*- adducts, respectively.

**a**

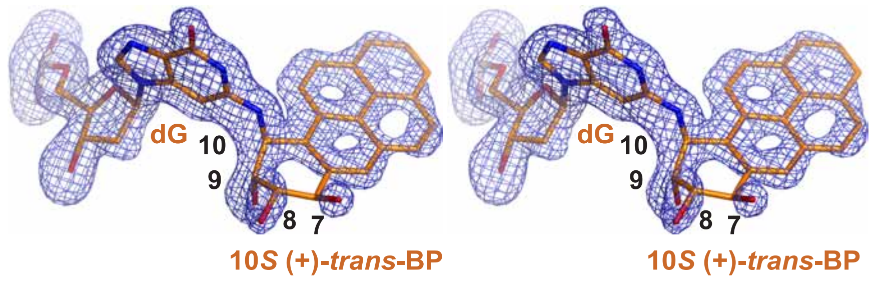


**b**

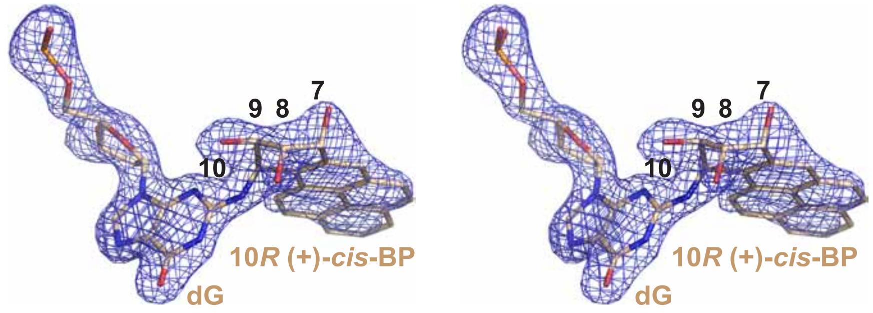


Supplementary Figure 2

**Supplementary Figure 2. Stereo views of the 10*S* (+)-*trans*- and 10*R* (+)-*cis*-BP-*N*$^2$-dG adducts in Rev1 ternary complexes.** (**a**) Stereo view of the 10*S* (+)-*trans*-BP-*N*$^2$-dG adduct. A simulated annealing Fo − Fc omit map (contoured at 3.0σ-level at 1.85 Å resolution and colored in blue) showing the clear electron density for the BP-moiety and the modified-dG. The dG and the BP C7-OH hydroxyl group are positioned above the BP benzylic ring, while the C9-OH and C8-OH hydroxyl groups are below, thus defining the conformation and the expected stereochemistry of the 10*S* (+)-*trans* BP benzylic ring. (**b**) Stereo view of the 10*R* (+)-*cis*-BP-*N*$^2$-dG adduct. A simulated annealing Fo − Fc omit map (contoured at 3.5σ at 1.92 Å resolution and colored in blue) showing clear density for the BP-moiety and the modified-dG. The dG as well as the C9-OH and C8-OH hydroxyl groups are oriented below the BP benzylic ring, while the C7-OH is above the ring. This confirms the expected stereochemistry of the 10*R* (+)-*cis* BP benzylic ring.

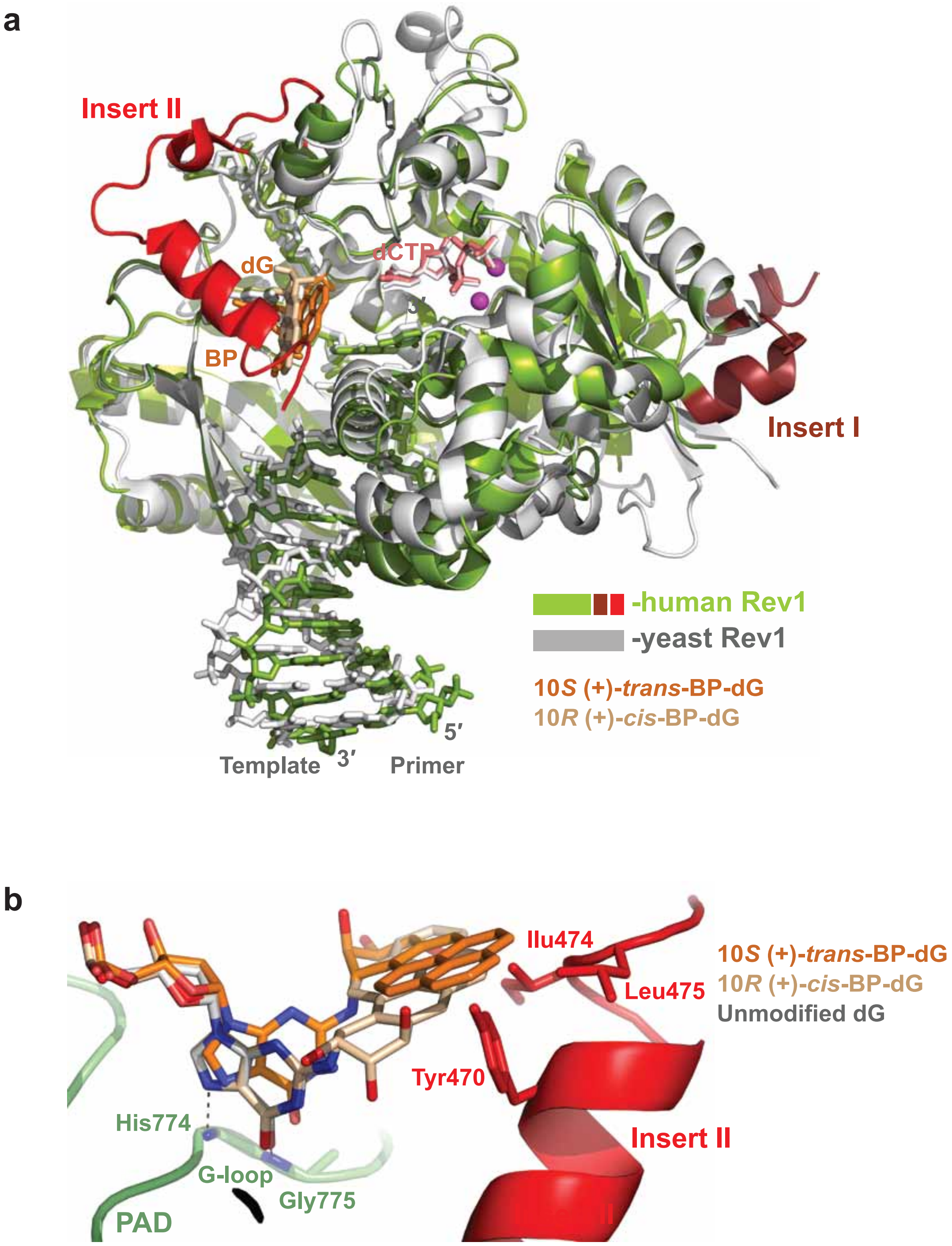


Supplementary Figure 3

**Supplementary Figure 3. Model of the 10*S* (+)-*trans*- and 10*R* (+)-*cis*-BP-dG modified human Rev1 complexes.** (**a**) Superposition of the overall structures of unmodified human Rev1 complex and 10*S* (+)-*trans*- and 10*R* (+)-*cis*-BP-dG containing yRev1 complexes. Unmodified-dG yeast Rev1 complex (shown in white) (PDB ID: 2AQ4)[35] is superimposed with the human Rev1 structure (PDB ID: 3GQC)[36] (shown in bright green) by the secondary structure topology alignment of the protein chains. The structures of the catalytic cores of human and yeast Rev1 are remarkably similar in spite of the fact that the proteins share a ~27 % sequence identity[36]. The distinct feature of the human Rev1 catalytic core is a number of large inserts including Inserts I and II into the Palm and Fingers domains, respectively, that are shown in dark and bright red colors. The 10*S* (+)-*trans*- and 10*R* (+)-*cis*-BP-dG adduct containing yeast Rev1 complexes are superimposed on the unmodified yeast Rev1 structure, with only the BP-dG moieties shown in orange (10*S* (+)-*trans*-BP-dG*)* and beige color (10*R* (+)-*cis*-BP-dG) sticks. The human Rev1 protein can accommodate both of these BP stereoisomeric adducts in the space between the PAD and the template−primer helix similar to the yeast protein. (**b**) Zoomed in view of the 10*S* (+)-*trans*-BP-dG and 10*R* (+)-*cis*-BP-dG adducts within the model of the BP-modified human Rev1 complexes. The hydrophobic side chains of Tyr470, Ile474 and Leu475 residues of the insert II of the human protein interact with the face and the edge of the hydrophobic BP pyrenyl ring systems and thus may help to stabilize the complexes. Only minor adjustments in the position of the side chain of Tyr470, possibly to an alternative rotamer, are required to avoid a minor steric clash with the 10*S* (+)-*trans*-BP moiety.
